# Investigation of the Incidence and Geographic Distribution of Bone and Soft Tissue Sarcomas in Canada: A National Population-Based Study

**DOI:** 10.3390/curroncol30060424

**Published:** 2023-06-09

**Authors:** Badria Alkazemi, Feras M. Ghazawi, François Lagacé, Vladimir Nechaev, Andrei Zubarev, Ivan V. Litvinov

**Affiliations:** 1 Faculty of Medicine, University of Leeds School of Medicine, Leeds LS2 9JT, UK; 2 Division of Dermatology, University of Ottawa, Ottawa, ON K1H 8M2, Canada; 3 Experimental Medicine Training Program, Department of Medicine, McGill University, Montreal, QC H4A 3J1, Canada; francois.lagace@mail.mcgill.ca; 4 Independent Researcher, 187015 Saint Peterburg, Russia; 5 Cancer Research Program, McGill University Health Centre Research Institute, McGill University, Montreal, QC H4A 3J1, Canada; 6 Division of Dermatology, Department of Medicine, McGill University, Montreal, QC H4A 3J1, Canada

**Keywords:** sarcoma, Kaposi sarcoma, bone sarcoma, axial sarcoma, peripheral sarcoma, soft tissue sarcoma, postal code, cancer, Canadian Cancer Registry, epidemiology, incidence, mortality, Canada

## Abstract

Sarcomas are a heterogeneous group of mesenchymal malignancies with various genetic and environmental risk factors. This study analyzed the epidemiology of sarcomas to gain insight into the incidence and mortality rates of these cancers in Canada, as well as to elucidate their potential environmental risk factors. Data for this study were obtained from le Registre Québécois du Cancer (LRQC) and from the Canadian Cancer Registry (CCR) for the period from 1992 to 2010. Mortality data were obtained from the Canadian Vital Statistics (CVS) database for the period from 1992 to 2010 using the International Classification of Diseases for Oncology, ICD-O-3, ICD-9, or ICD-10 codes, for all subtypes of sarcomas. We found that the overall sarcoma incidence in Canada decreased during the study period. However, there were select subtypes with increasing incidence. Peripherally located sarcomas were found to have lower mortality rates compared to axially located sarcomas, as expected. Clustering of Kaposi sarcoma cases in self-identified LGBTQ+ communities and in postal codes with a higher proportion of African-Canadian and Hispanic populations was observed. Forward Sortation Area (FSA) postal codes with a lower socioeconomic status also had higher Kaposi sarcoma incidence rates.

## 1. Introduction

Sarcomas are a diverse group of rare mesenchymal malignancies that can occur anywhere in the body and at any age [1]. Sarcomas can be categorized into two main groups, bone sarcomas and soft tissue sarcomas, with 80 to 90% of cases being soft tissue sarcomas [2,3]. Within these two categories, there are more than 100 histological subtypes [4]. The heterogeneity of sarcomas renders their epidemiological analysis challenging, and few studies have assessed the epidemiology of sarcomas collectively. To our knowledge, this is the first study to analyze and compare the incidence, mortality, and geographic localization of both bone and soft tissue sarcomas in Canada.

Fewer than 1% of malignancies in adults are sarcomas [1,5]. They are more common in the pediatric population, in which bone and soft tissue sarcomas account for 5% and 15% of cancers, respectively [1,6]. Despite their rarity, sarcomas carry a large disease burden in the pediatric population and are a significant cause of cancer deaths during the first 20 years of life [1,7]. In the United States, it is estimated that nearly 5500 Americans die from sarcomas each year [2]. 

There are numerous genetic syndromes that increase the risk of developing sarcomas, including neurofibromatosis type 1, McCune–Albright syndrome, Maffuci’s syndrome, and Li–Fraumeni syndrome [3,4,5,8,9]. Exposure to radiation is an established risk factor for sarcomas [1,3,10,11]. Those who received radiation therapy to treat childhood cancers had a 16-fold increased risk of developing sarcomas compared to those who did not [4]. Immune suppression has also been implicated in the development of sarcomas, notably Kaposi sarcoma in people diagnosed with AIDS [1,2]. 

There exist multiple environmental risk factors for sarcoma development. Water contaminated with radium at place of birth was found to be associated with sarcoma in Ontario, Canada, in a case–control study [12]. Fluoride in drinking water has been reported as a risk factor for the development of osteosarcoma [2]. However, other studies have found conflicting results regarding whether fluoride in drinking water could cause osteosarcoma [13]. In addition, patients whose parents worked on farms had an increased risk for pediatric Ewing’s sarcoma [14]. Various other chemical compounds implicated in the development of sarcomas include polyvinyl chloride (PVC), inorganic arsenic, anabolic steroids, and benzophenone, to name a few [1,4]. Occupational exposures that are associated with an increased sarcoma risk include herbicides [3,15,16], phenoxyacetic acid [3], and dioxins from industrial waste incinerators [2]. However, these associations were not observed in some studies in the United States and Sweden [4,17]. Many of the occupational risk factors are not fully established/confirmed because dose dependence has not been observed [2,4,18]. Additionally, many studies have small sample sizes and risk factor associations may have occurred by chance [19]. Several studies have hypothesized potential risk factors for sarcomas, but drawing definitive conclusions regarding the association between risk factors and sarcomas has proven to be challenging [2]. 

Globally, the incidence of sarcomas, particularly soft tissue sarcomas, is increasing. However, survival outcomes have remained unchanged over the past three decades. In England, the 5-year survival rate of osteosarcoma has not improved since the 1980s and the average survival has remained under 45% [20]. In the United States, soft tissue sarcomas had a mortality rate of up to 50% between 1978 and 2001 as per the Surveillance, Epidemiology, and End Results (SEER) Program database [1]. Therefore, the purpose of this study is to analyze the epidemiology of sarcomas to gain insight into the incidence and mortality rates of sarcomas in Canada, as well as to elucidate potential environmental risk factors.

## 2. Materials and Methods

This study was conducted in accordance with the CISSRDC-668035 and 13-SSH-MCG-3749 protocols approved by the Social Sciences and Humanities Research Council of Canada and the Québec Inter-University Centre for Social Statistics, respectively. In addition, in accordance with institutional policy, this study received an exemption from the McGill University Research Ethics Board review. This study was conducted following the same methods as previously reported [21,22,23,24,25]. Incidence data were obtained from the le Registre Québécois du Cancer (LRQC) for cases occurring in the province of Quebec and from the Canadian Cancer Registry (CCR) for cases occurring outside the province of Quebec. As sarcomas are heterogenous and rare, we categorized them into subtypes as per the ICD-O-3 classification, as shown in Supplementary Table S1. For example, muscle sarcoma includes rhabdomyosarcoma and leiomyosarcoma. In this study, only data for invasive cancers were analyzed. The high-level accuracy of the CCR/LRQC databases with respect to case ascertainment, tumor detection, and confirmation rate had been previously highlighted [23]. This study was limited to the analysis of data between 1992 and 2010 because the LRQC only includes data until 2010. Mortality data were obtained from the Canadian Vital Statistics (CVS) database for 1992–2010 using the International Classification of Diseases (ICD)-9 and ICD-10 codes [23]. Specifically, the International Statistical Classification of Diseases and Related Health Problems, 9th revision (ICD-9) was used for deaths occurring during 1992 through 1999 and the corresponding 10th revision (ICD-10) was used for deaths occurring from 2000 through 2010. 

The data available from the registries included in the analyses were the diagnostic data (ICD-O-3 codes), mortality data (ICD-9 or -10 codes), geographic data, and demographic data of those afflicted with each type of sarcoma. This included the type of sarcoma, year of diagnosis, sex, age at diagnosis, province, city, and Forward Sortation Area (FSA, or first 3 entries in a postal code) of residence. In particular, the incidence data and 95% confidence intervals (CI) based on national population counts, as well as data by province, by city, and by FSA, were obtained from the Statistics Canada’s Census of Population for the years 1996, 2001, 2006, and 2011. The provincial incidence rates were age-standardized to the Canadian population for comparison with the national incidence rate for the following sarcomas: fibrous sarcoma, muscle sarcoma, liposarcoma, and Kaposi sarcoma. This enabled us to obtain the age-standardized incidence rate (ASIR) [26]. The reason these subtypes were further analyzed was because they were the four subtypes with the highest incidence rates other than sarcoma not otherwise specified (NOS). Similarly, mapping analysis was completed on the four subtypes with the highest incidence excluding sarcoma NOS. The FSAs with a statistically significant higher incidence compared to the national rate were mapped using a geographic information system software (Tableau 10.3 from Tableau Software, Seattle Washington, USA) [27]. Statistical and mapping analysis was completed using the same methodology as previously described [21,22,23,24,25]. As individual sarcoma subtypes are rare, the crude data were reported as “per million individuals” as opposed to the conventional “per 100,000 individuals”. 

Analysis of the ethnic and socioeconomic status (SES) composition of each FSA was completed for Kaposi sarcoma. Ethnicity data were obtained from the 2001 and 2006 Canadian Censuses of Population. Each FSA was assigned to a quintile based on the percentage of its population that is Hispanic or African Canadian. The FSAs having the lowest percentage of Hispanics/African Canadians were placed in the first quintile (Q1), and those with the highest percentage were placed in the fifth quintile (Q5). Each FSA was also assigned to a quintile (Q1 to Q5) based on its average median income, which was used to represent SES. The first quintile (Q1) represents the FSAs with the lowest SES, whereas the fifth quintile (Q5) represents the FSAs with the highest SES. The incidence rate ratios and their corresponding 95% CI were calculated to compare the quintiles. 

## 3. Mandatory Data Rounding 

As the databases collected sensitive information, a number of steps were required to be taken to ensure that confidentiality is respected. The incidence and mortality data where the counts were ≥1 but <5 were not released as per the Social Sciences and Humanities Research Council (SSHRC) regulations. The data where 0 cases were reported and analyzed. In addition, the data from the CCR, LRQC, and CVS was randomly rounded to absolute numbers prior to analysis. These data were subsequently rounded to the nearest 5, regardless of whether it was lower or higher, to ensure compliance with the SSHRC to further protect patient confidentiality. This rounding scheme was unbiased, random, and independent of the other data. Any data that were rounded to the nearest 5 is indicated where necessary. 

## 4. Results

### 4.1. Incidence of Sarcomas in Canada between 1992 and 2010

In total, 25,895 Canadians were diagnosed with sarcomas between 1992 and 2010. The mean crude national incidence rate for sarcomas in Canada was 43.84 (95% CI 43.31–44.38) cases per million individuals per year throughout the 19-year span. The soft tissue and bone sarcoma subtypes with the highest annual incidence rates were muscle sarcoma and chondrosarcoma, respectively. The incidence rate for each recognized subtype is summarized in Table 1.

There was a slightly higher incidence of sarcomas in males compared to females (55% vs. 45%). The male-to-female ratio for sarcomas was 1.22:1. The subtypes with a higher predisposition in females were muscle sarcoma, unknown sarcoma, vascular sarcoma, and sarcomas not otherwise specified (NOS) (Table 2). The mean age at diagnosis ± standard deviation for all sarcomas collectively was 53.59 ± 21.73 years. This was further assessed for each subtype of sarcoma (Table 3). Notably, the mean age of diagnosis of Ewing’s sarcoma was 21.99 ± 14.76 years because it is mainly seen in the pediatric population. Kaposi sarcoma also had a younger mean age at diagnosis of 46.00 ± 15.57 years as it is often seen in younger, sexually active patients who had contracted HIV. Overall, 11,240 patients (44%) were diagnosed at the age of 60 or above, 7915 patients (31%) were diagnosed between the ages of 40 and 59 years, and 6480 patients (25%) were diagnosed at an age younger than 40 years. 

**Figure 1:**
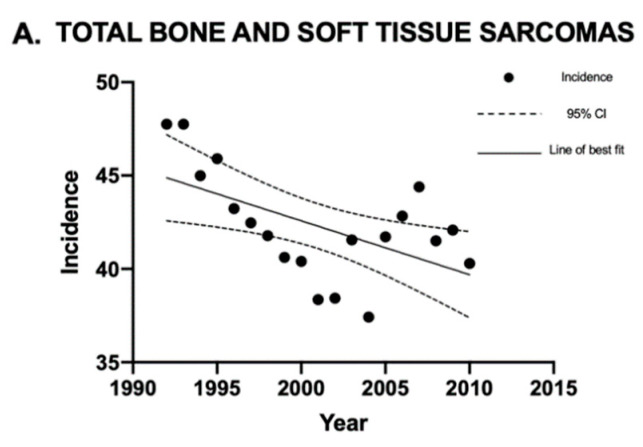

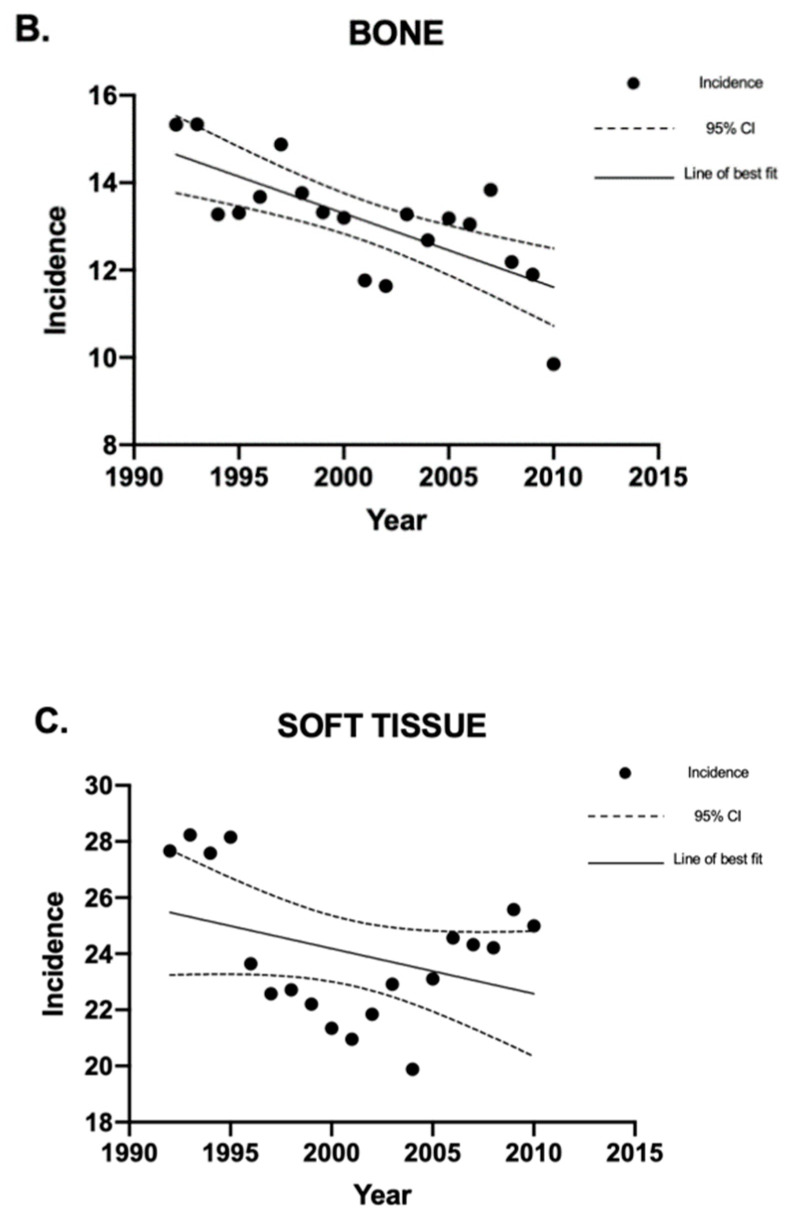
Crude incidence rates of all sarcomas, bone sarcomas, and soft tissue sarcomas in Canada between 1992 and 2010. Linear regression analysis of incidence rates for all sarcomas, bone sarcomas, and soft tissue sarcomas, expressed as cases per 1 million individuals per year, between 1992 and 2010. The data points indicate incidence for a given year, the solid lines indicate the line of best fit, and the dotted lines indicate the upper and lower 95% CI. Coefficient of determination is expressed as [R^2^]. Statistical significance is expressed as *p*-values. (**A**) Regression analysis for the incidence of bone and soft tissue sarcomas combined: [R^2^] = 0.31, *p*-value = 0.013, and the slope of the line is −0.29. (**B**) Regression analysis for the incidence of all bone sarcomas: [R^2^] = 0.51, *p*-value = 0.0005, and the slope of the line is −0.17. (**C**) Regression analysis for the incidence of soft tissue sarcomas: [R^2^] = 0.13, *p*-value = 0.127, and the slope of the line is −0.16. “CI” stands for “confidence interval”.

**Figure 2:**
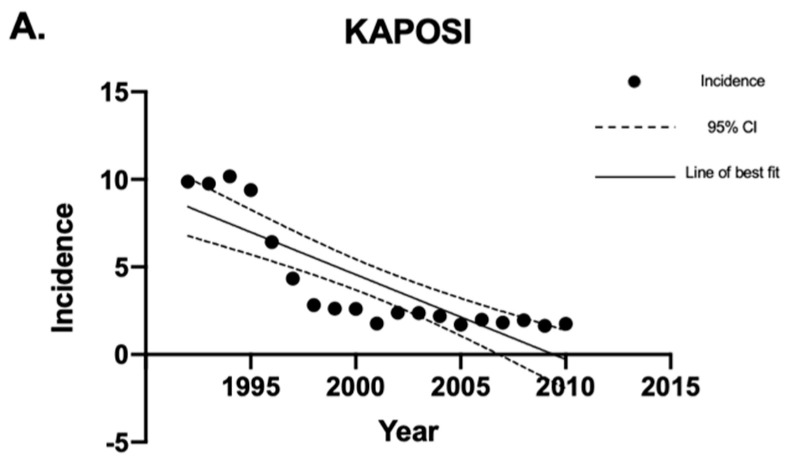

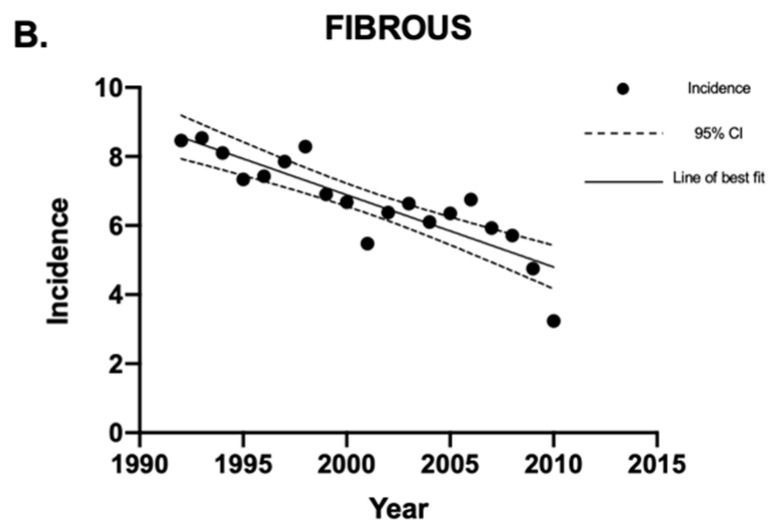
Crude incidence rates of Kaposi and fibrous sarcomas in Canada between 1992 and 2010. Linear regression analysis of incidence rates of Kaposi and fibrous sarcoma, expressed as cases per 1 million individuals per year, between 1992 and 2010. The data points indicate incidence for a given year, the solid lines indicate the line of best fit, and the dotted lines indicate the upper and lower 95% CI. Coefficient of determination is expressed as [R^2^]. Statistical significance is expressed as *p*-values. (**A**) [R^2^] = 0.71; *p* < 0.0001; and the slope of the line is −0.48 cases per million individuals per year. (**B**) [R^2^] = 0.76; *p* < 0.0001; and the slope of the line is −0.21 cases per million individuals per year. “CI” stands for “confidence interval”.

**Figure 3:**
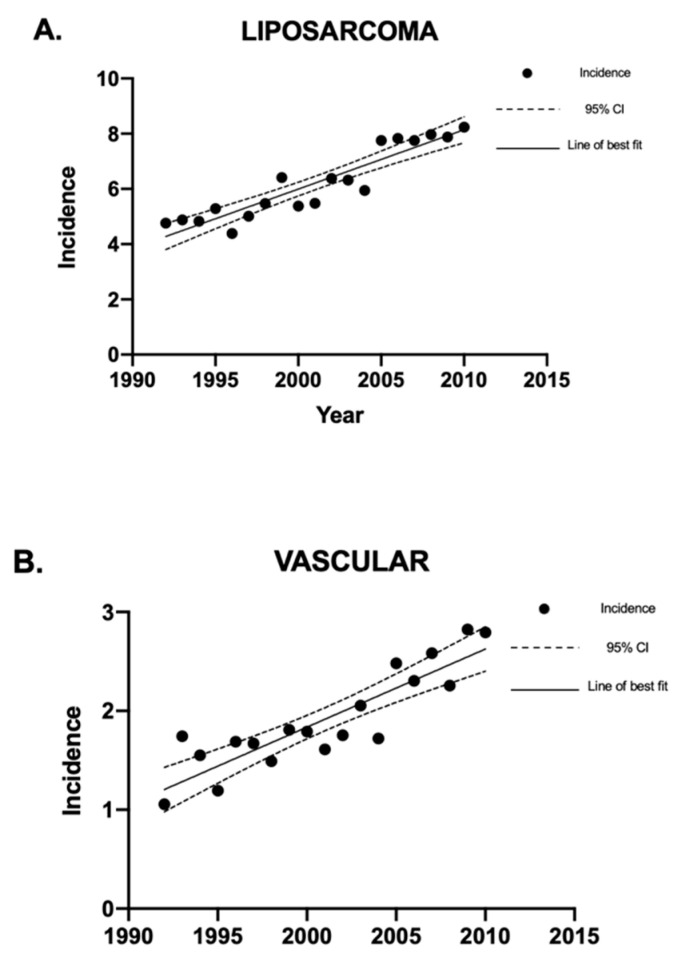
Crude incidence rates of liposarcoma and vascular sarcomas in Canada between 1992 and 2010. Linear regression analysis of the incidence rates of liposarcoma and vascular sarcoma, expressed as cases per 1 million individuals per year, between 1992 and 2010. The data points indicate incidence for a given year, the solid lines indicate the line of best fit, and the dotted lines indicate the upper and lower 95% CI. Coefficient of determination is expressed as [R^2^]. Statistical significance is expressed as *p*-values. (**A**) [R^2^] = 0.86; *p* < 0.0001; and the slope of the line is 0.21 cases per million individuals per year. (**B**) [R^2^] = 0.78; *p* < 0.0001; and the slope of the line is 0.079 cases per million individuals per year. “CI” stands for “confidence interval”.

Linear regression analysis was used to analyze the sarcoma incidence trends over time. Overall, the incidence of sarcomas in Canada decreased between 1992 and 2010 (Figure 1). The sarcoma subtypes with a statistically significant (*p* < 0.05) decrease in crude incidence rates were Kaposi sarcoma and fibrous sarcoma (Figure 2). The sarcomas with a statistically significant (*p* < 0.05) increase in crude incidence rates were liposarcoma, vascular sarcoma, sarcoma NOS, chondrosarcoma, synovial sarcoma, dermatological sarcoma, and notochordal sarcoma. Liposarcoma had the largest increase in crude incidence rate, followed by vascular sarcoma (Figure 3).

### 4.2. Mortality of Sarcomas in Canada between 1992 and 2010

The mortality rates of bone and soft tissue sarcomas were analyzed using the ICD-9 or ICD-10 codes (Table 4). Sarcoma NOS was omitted from the analysis as neither subtype nor location could be specified. The mortality rates of axial and peripheral sarcomas were compared, and the results are summarized in Table 5.

#### 4.2.1. Bone Sarcoma Mortality

**Figure 4:**
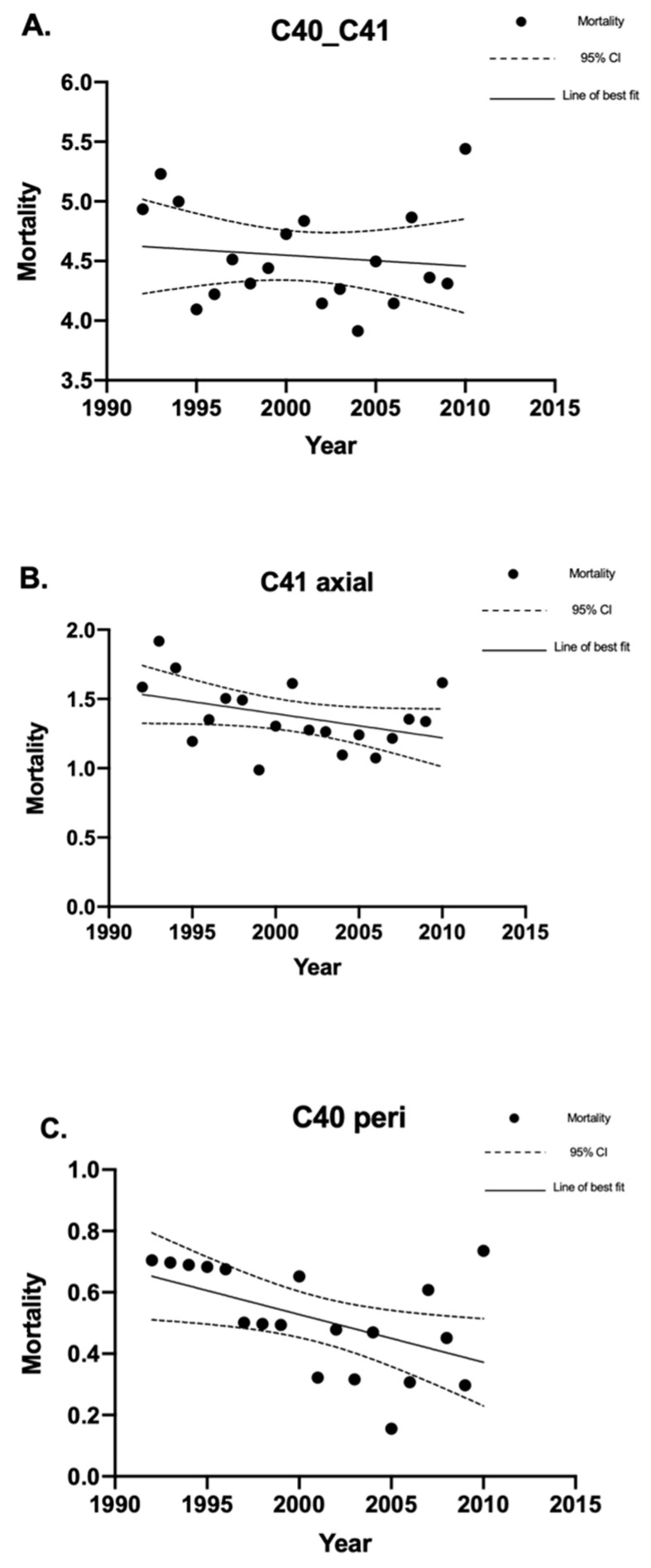
Crude mortality rates of bone sarcomas in Canada between 1992 and 2010. Linear regression analysis of the crude mortality rates of bone sarcoma, expressed as deaths per 1 million individuals per year, between 1992 and 2010. The data points indicate the mortality rate for a given year, the solid lines indicate the line of best fit, and the dotted lines indicate the upper and lower 95% CI. Coefficient of determination is expressed as [R^2^]. Statistical significance is expressed as *p*-values. (**A**) Crude mortality rates for all deaths due to bone sarcoma (C40_41): [R^2^] = 0.015; *p* = 0.61; and the slope of the line is −0.0091 deaths per million individuals per year. (**B**) Crude mortality rates for all deaths due to axial bone sarcoma (C41 axial): [R^2^] = 0.17; *p* = 0.082; and the slope of the line is −0.017 deaths per million individuals per year. (**C**) Crude mortality rates for all deaths due to peripheral bone sarcoma C40 (peripheral): [R^2^] = 0.26; *p* = 0.026; and the slope of the line is −0.016 deaths per million individuals per year. “CI” stands for “confidence interval”.

In total, there were 2675 deaths from bone sarcomas. The crude mortality rate for all bone sarcomas was 4.53 (95% CI 4.35–4.70) deaths per million individuals per year. This included defined/specified peripheral and axial bone sarcoma mortality, as well as deaths from bone sarcomas with no specified location (Figure 4A). There were 795 deaths from axial bone sarcomas, which had a crude mortality rate of 1.34 (95% CI 1.25–1.44) deaths per million individuals per year (Figure 4B). There were 295 deaths from defined/specified peripheral bone sarcomas, which had a crude mortality rate of 0.50 (95% CI 0.44–0.56) deaths per million individuals per year (Figure 4C). The remaining 1585 deaths did not have a specified location, whether axial or peripheral. 

The mortality rates for bone sarcomas overall did not change significantly between 1992 and 2010. However, there was a decrease in mortality for peripheral bone sarcomas (*p* = 0.026). The male-to-female ratio for bone sarcoma mortality was 1.37:1. Peripheral bone sarcoma mortality had a bimodal age distribution, with peaks between the ages of 10 and 19 years and between the ages of 70 and 79 years. Axial bone sarcoma did not show a bimodal distribution but demonstrated an increased mortality rate with increasing age until the ages of 70–79 and decreased thereafter. The overall mean age of bone sarcoma mortality was 53.1 ± 25.4 years. The mean age of mortality for specified peripheral and axial bone sarcomas was 54.5 ± 26.3 and 65.3 ± 19.5 years, respectively. 

#### 4.2.2. Soft Tissue Sarcoma Mortality

**Figure 5:**
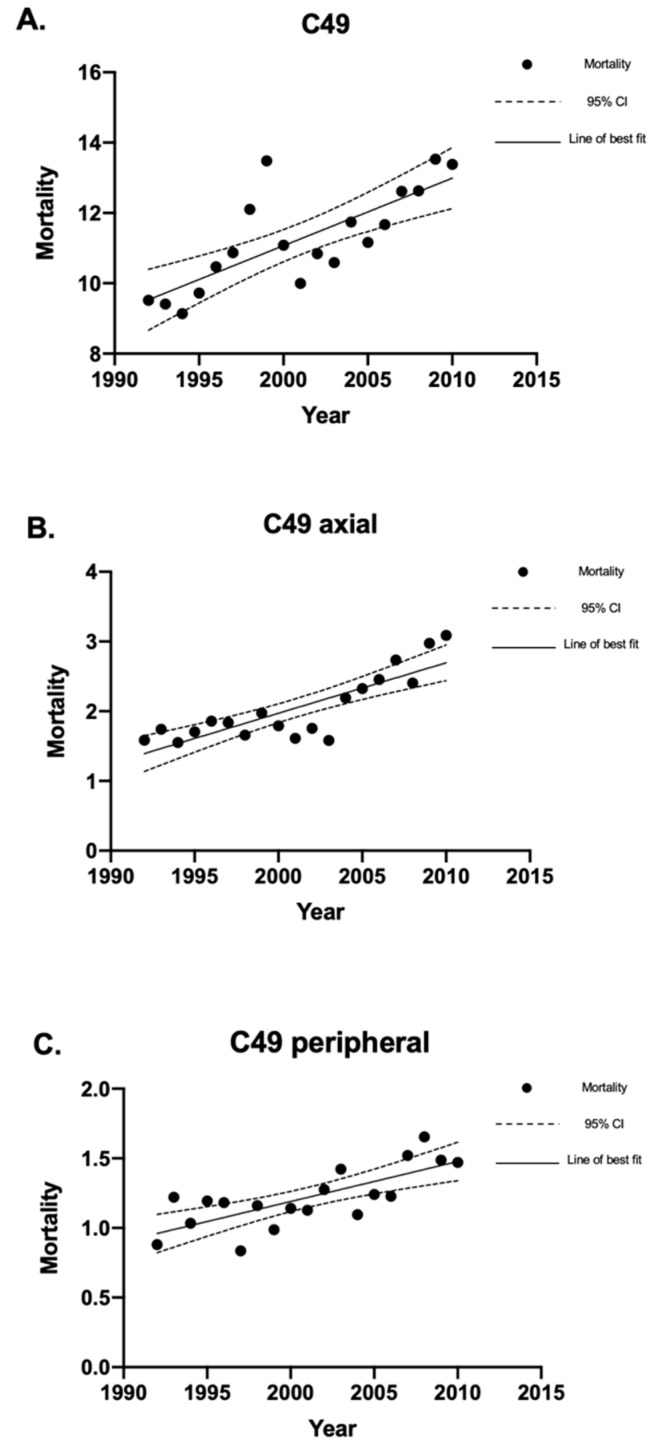
Crude mortality rates of soft tissue sarcomas in Canada between 1992 and 2010. Linear regression analysis of the crude mortality rates of soft tissue sarcoma, expressed as deaths per 1 million individuals per year, between 1992 and 2010. The data points indicate mortality rate for a given year, the solid lines indicate the line of best fit, and the dotted lines indicate the upper and lower 95% CI. Coefficient of determination is expressed as [R^2^]. Statistical significance is expressed as *p*-values. (**A**) Crude mortality rates for all soft tissue sarcomas: [R^2^] = 0.59; *p* = 0.0001; and the slope of the line is 0.19 deaths per million individuals per year. (**B**) Crude mortality rates for axial soft tissue sarcomas: [R^2^] = 0.69; *p* < 0.0001; and the slope of the line is 0.072 deaths per million individuals per year. (**C**) Crude mortality rates for all peripheral soft tissue sarcomas: [R^2^] = 0.56; *p* = 0.0002; and the slope of the line is 0.029 deaths per million individuals per year. “CI” stands for “confidence interval”.

There were 6680 deaths from soft tissue sarcomas. The crude mortality rate for soft tissue sarcomas was 11.30 (95% CI 11.03–11.58) deaths per million individuals per year (Figure 5A). This included peripheral and axial soft tissue sarcoma mortality, as well as deaths from soft tissue sarcomas with no specified location. There were 1225 deaths from axial soft tissue sarcomas, which had a crude mortality rate of 2.07 (95% CI 1.96–2.19) deaths per million individuals per year (Figure 5B). Peripheral soft tissue sarcomas had 715 deaths, which had a crude mortality rate of 1.21 (95% CI 1.12–1.30) deaths per million individuals per year (Figure 5C). The remaining 4740 deaths did not have a specified location, whether axial or peripheral. 

An Increase In the crude mortality rate for soft tissue sarcomas was observed between 1992 and 2010. The male-to-female ratio for soft tissue sarcoma mortality was 1.02:1. The mortality rate for soft tissue sarcomas increased with age until the age group of 70–79 and decreased thereafter. The mean mortality age for all soft tissue sarcomas was 61.5 ± 19.8 years. The mean age of mortality was 66.9 ± 19.8 years for specified peripheral soft tissue sarcomas and 65.6 ± 18.4 years for specified axial soft tissue sarcomas. 

### 4.3. Geographic Analysis

#### 4.3.1. Geographic Distribution of Fibrous Sarcoma Cases

**Figure 6:**
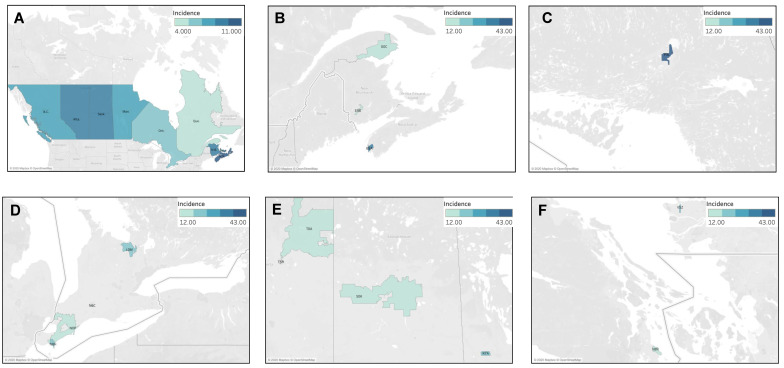
Geographic distribution of fibrous sarcoma cases (**A**) by province, highlighting individual FSAs with statistically significant high incidence in (**B**) Quebec/Atlantic Canada, (**C**) northern Ontario, (**D**) southwestern Ontario, (**E**) Prairie provinces, and (**F**) British Columbia.

The national incidence rate of fibrous sarcoma was 6.6 cases per million individuals per year. The provinces of Nova Scotia, New Brunswick, Alberta, Saskatchewan, and British Columbia were found to have significantly higher ASIR than the national average, with the highest being in Nova Scotia at 10.28 cases per million individuals per year (95% CI 8.85–11.89). Conversely, Quebec had a significantly lower incidence rate of 4.95 cases (95% CI 4.59–5.33) (Figure 6A). The distribution of fibrous sarcoma cases was then analyzed by FSA postal code, and the results are presented in Figure 6B–F. Of the 17 statistically significant high-incidence FSAs identified, 29% (5/17) were located in Ontario, 24% (4/17) in Alberta, 12% (2/17) each in Quebec and British Columbia, and, finally, 6% (1/17) each in Nova Scotia, New Brunswick, Manitoba, and Saskatchewan. Clustering of large groups of postal code regions was not observed; however, several FSAs with very high incidence were noted. The first corresponded to the FSA P3L, representing Greater Sudbury in Northern Ontario with a rate of 42.68 cases per million individuals per year (95% CI 15.93–91.98), showing an incidence ~6.5 times higher than the Canadian average. The FSAs of B5A in Yarmouth (Nova Scotia), T5H and T5A in Edmonton (Alberta), and V5Z in Vancouver (British Columbia) also had significantly high incidence rates. 

#### 4.3.2. Geographic Distribution of Kaposi Sarcoma Cases

**Figure 7:**
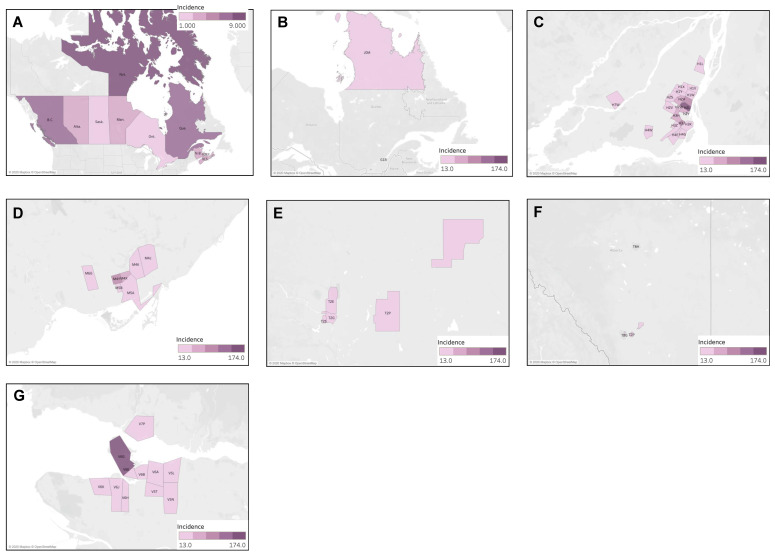
Geographic distribution of Kaposi sarcoma cases (**A**) by province, highlighting individual FSAs with statistically significant high incidence in (**B**) Quebec, (**C**) Montreal, (**D**) Toronto, (**E**) Calgary, (**F**) Alberta (including Edmonton and Calgary), and (**G**) Vancouver.

The national incidence rate of Kaposi sarcoma was 3.9 cases per million individuals per year. The provinces of British Columbia and Quebec were found to have significantly higher ASIR than that of the national average, at 7.30 (95% CI 6.71–7.93) and 6.42 (95% CI 6.01–6.85) cases per million individuals per year, respectively. Conversely, Alberta, Ontario, and Saskatchewan had significantly lower incidence rates, with the lowest being in Saskatchewan at 1.33 (95% CI 0.86–1.95) cases per million individuals per year (Figure 7A). The distribution of cases of Kaposi sarcoma was then analyzed by FSA, as presented in Figure 7B–G. Of the 55 statistically significant high-incidence FSAs identified, 49% (27/55) were located in Quebec, 22% (12/55) in British Columbia, 15% (8/55) in Ontario, 11% (6/55) in Alberta, and, finally, 2% (1/55) in New Brunswick. The highest incidence was observed in the FSA V6E, representing Davie Village (self-identified LGBTQ+ community) in Vancouver, with 174.00 (95% CI 136.75–218.26) cases per million individuals per year, showing an incidence ~45 times higher the Canadian average. High incidence clustering was concentrated in three urban areas: Vancouver, Montreal, and Toronto. In Vancouver, clustering was seen in Davie Village (V6E), the Downtown Eastside (V6A), and other areas of downtown Vancouver. In Toronto, clustering was observed in Church and Wellesley (self-identified LGBTQ+ community) (M4Y), and surrounding core downtown areas. Finally, in Montreal, clusters were seen in various downtown areas, including the Gay Village (H2L), Hochelaga-Maisonneuve (H1V, H1W), Plateau Mont-Royal (H2H), Rosemont Petite-Patrie (H1X, H1Y), Petite Bourgogne (H3J), Pointe-Saint-Charles (H3K), Ville-Émard (H4E), and Côte Saint-Luc Areas (H4W). Finally, the region of Nunavik (J0M) also demonstrated a significantly high incidence of Kaposi sarcoma, where classic variant of Kaposi Sarcoma is not uncommon.

#### 4.3.3. Geographic Distribution of Liposarcoma Cases

**Figure 8:**
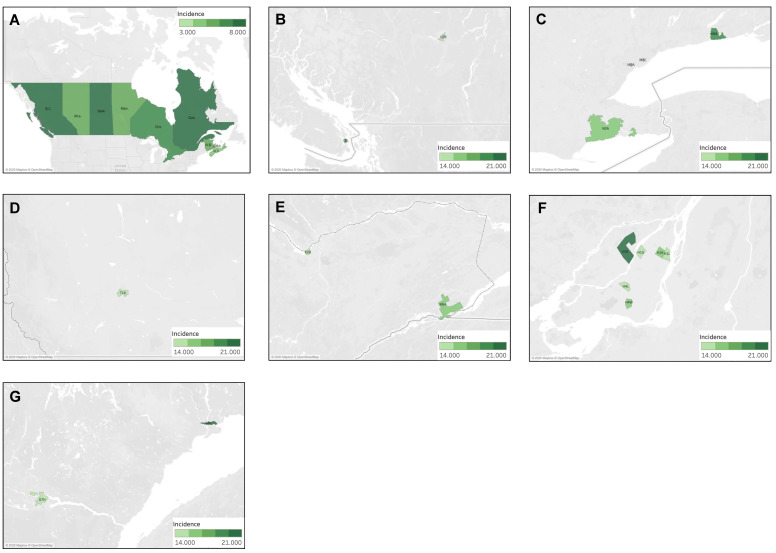
Geographic distribution of liposarcoma cases by (**A**) province, highlighting individual FSAs with statistically significant high incidence in (**B**) British Columbia, (**C**) Southern Ontario, (**D**) Alberta, (**E**) Eastern Ontario, (**F**) Montreal, and (**G**) Quebec.

The national incidence rate of liposarcoma was 6.3 cases per million individuals per year. The provinces of British Columbia and Quebec were found to have significantly higher ASIR than that of the national average, at 7.21 (95% CI 6.61–7.83) and 7.04 (95% CI 6.61–7.49) cases per million individuals per year, respectively. Conversely, Alberta had a significantly lower incidence rate of 5.10 (95% CI 4.54–5.71) (Figure 8A). The distribution of liposarcoma cases was then analyzed by FSA, as presented in Figure 8B–G. Of the 17 statistically significant high-incidence FSAs identified, 47% (8/17) were located in Quebec, 35% (6/17) in Ontario, 12% (2/17) in British Columbia, and, finally, 6% (1/17) were in Alberta. The highest incidence was observed in the FSA G5C, corresponding to Baie-Comeau in Quebec, with 20.76 cases per million individuals per year (95% CI 6.77–48.34), showing an incidence ~3 times higher the Canadian average. High incidence clustering was observed in Montreal in the regions of Duvernay, Anjou, Mercier, Montreal North, Saint-Laurent, and Côte Saint-Luc. Isolated high-incidence regions were also observed in Sidney (V8L, British Columbia), Cobourg (K9A, Ontario), and along the St. Lawrence River and Haldimand County (N0A, Ontario) which is a rural region bordering Lake Eerie.

#### 4.3.4. Geographic Distribution of Muscle Sarcoma Cases

**Figure 9:**
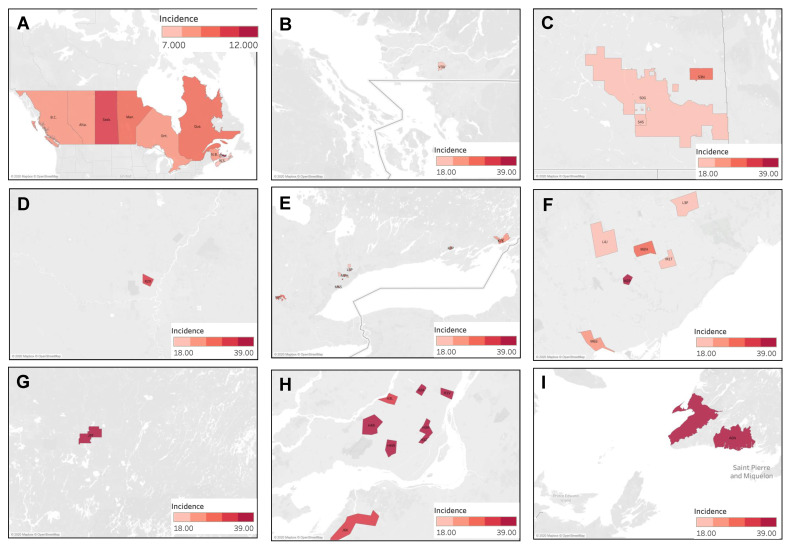
Geographic distribution of muscle sarcoma cases by (**A**) province, highlighting individual FSAs with statistically significant high incidence in (**B**) British Columbia, (**C**) Saskatchewan, (**D**) Manitoba, (**E**) Ontario, (**F**) Toronto, (**G**) Quebec, (**H**) Montreal, and (**I**) Newfoundland and Labrador.

The national incidence of muscle sarcoma was 10.4 cases per million individuals per year. No provinces were found to display a significantly higher incidence rate than the Canadian average. The provinces of Quebec, Ontario, Alberta, British Columbia, and Nova Scotia displayed significantly lower incidence rates, with the lowest being in Nova Scotia at 7.38 cases (95% CI 6.17–8.76) (Figure 9A). The distribution of muscle sarcoma cases was then analyzed by FSA, and the results are presented in Figure 9B–I. Of the 25 statistically significant high-incidence FSAs identified, 40% (10/25) were located in Ontario, 36% (9/25) in Quebec, 12% (3/25) in Saskatchewan, and, finally, 4% (1/25) each in Newfoundland and Labrador, Manitoba, and British Columbia. The highest incidence was observed in the FSA J9T, corresponding to Amos in Quebec, with 38.26 cases per million individuals per year (95% CI 18.47–70.05), showing an incidence ~4 times higher than the Canadian average. High-incidence clustering was noted in Montreal, Quebec, in Saint-Laurent (H4R), Côte Saint-Luc (H4W), downtown southwest areas (H3Z, H3H), Ahunstic (H3L), Saint-Michel (H2A), Maisonneuve (H1V), and Chateauguay (J6K). Clustering was also observed in Toronto, Ontario, in North York (M2P, M2H), Scarborough (M1T), West Toronto (M6S), Thornhill (L4J), and Markham (L3P). In Saskatchewan, clustering was noted in Yorkton (S3N), Regina (S4S), and rural regions surrounding Regina (S0G). Finally, higher incidence was noted in rural Newfoundland and Labrador in the southwest Port au Port Peninsula (A0N). 

### 4.4. Analysis by Ethnicity and Socioeconomic Status

There was a significant association between Kaposi sarcoma incidence rates and socioeconomic status (SES). There were no reported cases of Kaposi sarcoma in Q5_SES_, representing all FSAs with an average median household income greater than CAD 35,000. Furthermore, the incidence rates were significantly lower in the second highest SES quintile (Q4) compared to the lowest SES quintile (Q1) (IRR_SES Q4 vs. Q1_ = 0.26; 95% CI 0.19–0.36) (Table 6). Regarding ethnicity, in general, incidence rates were significantly higher in the quintiles with a higher percentage of African Canadian and Hispanic individuals compared to the quintiles with a lower percentage of African Canadian and Hispanic individuals. Table 7 and Table 8 present the incidence rate ratios comparing the various quintiles to quintile 1 (Q1) for African Canadian and Hispanic ethnicities, respectively. In particular, Q4_African-Candian_ and Q5_African-Canadian_ both had significantly greater incidence rates than Q1_African-Canadian_ and Q2-Q5_Hispanic,_ all of which had significantly greater incidence rates than Q1_Hispanic_. 

## 5. Discussion

To our knowledge, this is the first study to analyze the epidemiology and mortality of bone and soft tissue sarcomas in Canada. The crude national incidence rate for all sarcomas was 43.84 (95% CI 43.31–44.38) cases per million individuals per year. This was lower than the incidence of sarcomas in Europe and the United States. The incidence of sarcomas in the United States between 2002 and 2014 was 71 cases per million individuals per year [28]. An increase in incidence was observed from ~68 cases to 77 cases per million individuals per year between 2002 and 2014 [28]. The crude incidence of sarcomas in Europe between 1995 and 2002 was 56 cases per million individuals per year [3]. In France, the crude incidence of soft tissue sarcomas alone was 62 cases per million individuals per year [29]. A slight predisposition to have sarcomas in males was observed in this study, which was also observed in other European studies [3,4,20]. In a study in northern England, it was observed that males had a higher incidence of bone sarcomas as a whole, as well as a higher incidence of its subtypes in the 0–39 age group [7]. However, in another study, the male predisposition to have sarcomas was only statistically significant for the bone sarcoma subtypes [4]. The RARECARE project, which assessed data from 76 population-based cancer registries, found that soft tissue sarcomas had an incidence rate of ~47 cases per million individuals per year [3]. One study analyzed the incidence of soft tissue sarcomas in Australia between 1982 and 2009. It was found that the incidence increased, with the ASIR changing from 47 to 58 cases per million individuals per year between 1982 and 2009 [30]. The median age at diagnosis was also found to have increased from 58 to 63 years between 1982 and 2009 [30]. A recent study based in France analyzed the incidence of all types of sarcomas and found that the incidence ranged from 10 cases to less than 0.1 cases per million individuals per year, depending on the subtype of sarcoma [31]. However, it was found that the combined incidence of all sarcomas and connective tumors of intermediate malignancy amounted to nearly 95.1 cases per million individuals per year [31]. This study found that, by assessing recent data from 2013 to 2016, the incidence was higher than previously reported. It remains challenging to compare the true incidence of all sarcomas combined, or bone and soft tissue sarcomas separately, to other countries. Different countries maintain various population-based cancer registries spanning different years/periods. Additionally, inclusion/exclusion criteria vary significantly and at times do not comply with the International Agency for Research on Cancer (IARC) standards. Several studies have highlighted the challenges in assessing the incidence and mortality rates for such heterogenous tumors.

There was a statistically significant decrease in the incidence of all bone sarcomas between 1992 and 2010. There was also a statistically significant decrease in mortality from peripheral bone sarcomas, as well as mortality from all soft tissue sarcomas, peripheral soft tissue sarcomas, and axial soft tissue sarcomas. We found different mortality outcomes depending on the location of the sarcoma, as expected, since surgical management of peripheral sarcomas is more feasible when compared to axial sarcomas. In Canada, peripheral bone sarcomas had nearly half the mortality rate of axial bone sarcomas. Peripheral soft tissue sarcomas also had a lower mortality rate compared to axial soft tissue sarcomas. Similarly, in Sweden and Norway, peripheral sarcomas had nearly double the survival rates compared to axial tumors (70% vs. 35%) [32]. Furthermore, axial sarcomas may occur in the retroperitoneal space where tumors are able to grow to large sizes before patients exhibit symptoms. Therefore, patients who have axial tumors, particularly in the retroperitoneum, present with more advanced stages of cancer, thereby reducing the possibility of complete resection and, subsequently, leading to poor disease prognosis [33]. 

The most common subtype of sarcoma was muscle sarcoma, representing 24% of sarcomas in Canada. This is in keeping with the European RARECARE project, which also found muscle sarcoma to be the most common sarcoma and represented 20% of all sarcoma cases in Europe [3]. In England and the United States, leiomyosarcoma, a type of muscle sarcoma, caused 22% and 24% of sarcomas, respectively [4,34]. 

In our geographic analysis, a higher incidence of Kaposi sarcoma was seen in FSAs delineating self-identified LGBTQ+ communities, including Davie Village (V6E) in Vancouver [35], Church and Wellesley (M4Y) in Toronto [36], and Gay Village (H2L) in Montreal [37]. Kaposi sarcoma incidence was also higher in FSAs with lower SES, including Downtown Eastside (V6A) in Vancouver. This postal code is known for its high intravenous drug use and is known as “Canada’s poorest postal code” [38]. Similar findings were also observed in the United States, where Kaposi sarcoma incidence was higher in areas with lower SES [39]. According to our analysis, FSAs with a predominant African Canadian and Hispanic populations had a higher incidence of Kaposi sarcoma. Similarly, Hsieh et al. found that Kaposi sarcoma was more common in African American and Hispanic males compared to Caucasian males in the United States [40].

Clustering of higher-incidence FSAs for liposarcomas was observed in the Quebec and Ontario regions bordering the St. Lawrence River. Environmental endocrine-disrupting chemicals, such as bisphenol A (BPA) and Estradiol-17b, that are commonly used in pharmaceutics and industrial production are unfortunate ecological waste products. According to the 2017 Fresh Water Quality Monitoring and Surveillance report from Environment and Climate Change Canada, estradiol-17b levels were considerably elevated throughout different measurement points along the St. Lawrence River. Another study found increased levels of estradiol-17b in wastewater treatment plants in Ontario [41]. With over 90% of Montreal’s drinking water coming from the St. Lawrence River, this finding emphasizes the need to examine environmental exposure as an important element in the pathogenesis of sarcomas. Estradiol-17b has been demonstrated to induce transformation and tumorigenesis in human breast epithelial cells [42,43] and ovarian cancer cell lines [44] and plays role in the feminization of lipid metabolism in marine life [45,46]. Currently, BPA and Estradiol-17b are not considered risk factors in the pathogenesis of sarcomas. Additional studies will be necessary to provide answers as to whether endocrine-disrupting chemicals may cause sarcomas. 

In the FSAs in British Columbia, fibrous and muscle sarcomas predominated sarcoma incidence. In this study, muscle sarcomas were the most common sarcomas in patients aged 40–79, while fibrous sarcomas were the most common soft tissue sarcomas in patients over the age of 80 years old. Data by FSA was not age-standardized due to the rarity of sarcomas. In the 2006 census, an estimated 63–80% of the population in these FSAs were aged 65 years and over [47]. Therefore, the higher incidence of sarcomas in these FSAs might be linked in-part to the aging population. 

This study had several limitations. Notably, the crude incidence and mortality rates were analyzed over the 19-year period. The crude rates were used due to the rarity of the sarcomas analyzed, which would make it not possible to perform analysis by FSA. Therefore, the findings in this study do not account for changes in the population structure over the study time period. 

Retrospective epidemiological studies are limited by the accuracy of the databases used. While measures were taken to ensure the quality of the data used in this study, there is still the risk of missing or misclassifying data. The incidence data were obtained from two separate databases that recorded information from different regions in Canada. Therefore, some results might be due to the variations in how the data were collected between the two databases. The rarity of sarcomas also means that many patients did not have their specific sarcoma subtype or location, whether axial or peripheral, accurately recorded. The heterogeneity of sarcomas makes it difficult to study each subtype in great detail while producing an overall picture of the trends in incidence and mortality rates for sarcomas as a whole. It is also difficult to elucidate risk factors applicable to all subtypes.

Within each type of sarcoma, multiple subtypes are outlined in Supplementary Table S1. Many of these subtypes of sarcomas are considered “ultra-rare sarcomas” because of their particularly low incidence [48]. These ultra-rare sarcomas contribute to 20% of the sarcoma incidence in a study that analyzed European and Asian populations [48]. This indicates that despite their rarity, they collectively contribute to a considerable extent of the sarcoma disease burden. However, this study did not assess the epidemiology of ultra-rare sarcomas separately. Future research on the epidemiology of ultra-rare sarcomas in Canada may elucidate valuable information that can contribute to the global effort to advance sarcoma knowledge and treatments. 

The incidence and mortality registries used in this study collected different information that could not be synthesized and analyzed together (i.e., ICD-O-3 for tumor incidence vs. ICD-9 or ICD-10 for causes of mortality). This posed a limitation as the CCR did not collect information on where sarcomas occurred (whether axial or peripheral). Therefore, it remains unknown whether the mortality trends (managed by the CVS), particularly when comparing axial and peripheral sarcoma mortality, were due to a higher or a lower incidence.

The findings of this study suggest a relationship between environmental risk factors and sarcoma incidence. However, a causal relationship between environmental exposures and sarcoma incidence cannot be established. There may be confounding factors that may explain the findings presented in this study. The heterogeneity and the rarity of sarcomas make it challenging to postulate environmental causes. In addition, by studying subtypes of sarcomas within larger groups, specific associations between rare subtypes may be masked by the larger groups of sarcomas (such as studying “muscle sarcoma” as a whole as opposed to studying spindle cell rhabdomyosarcoma) [16]. Nevertheless, this study can serve as a step for future studies that may unveil whether there is an environmental cause of sarcoma incidence.

## 6. Conclusions

In conclusion, this epidemiological study highlights that sarcoma incidence in Canada decreased overall during the study period. However, there were selected subtypes with increasing incidence. Mortality rates did not change significantly, but peripheral sarcomas were found to have lower mortality rates compared to axial sarcomas, as expected. Clustering of Kaposi sarcoma in self-identified LGBTQ+ communities and in postal codes with a high proportion of African Canadian and Hispanic populations was observed. FSAs with lower socioeconomic status also had higher incidence rates of Kaposi sarcoma. Grouping/clustering of high-incidence FSAs for other sarcomas was noted, and these results present intriguing avenues for future studies investigating extrinsic risk factors for these tumors. Definitive associations between environmental exposures and sarcomas are not possible at this stage of the study. However, this work may serve as an important foundation for future studies that investigate the relationships between exposures/risk factors and sarcomas in Canada. One of the challenges of epidemiological studies is to present findings of such a heterogenous group of cancers while attempting to identify potential environmental risk factors. However, it is imperative to highlight that dose–response association was not studied and, therefore, no conclusion can be derived regarding the effects of potential environmental risk factors on the epidemiological findings. This study can provide the foundation for future longitudinal studies to examine dose–response relationships between environmental risk factors and sarcomas. 

## Figures and Tables

**Table 1:** Average crude national incidence and 95% CI for each subtype of sarcoma between 1992 and 2010, expressed as ‘Per Million Individuals Per Year’.

Subtype	Incidence	95% CI
Muscle Sarcoma *	10.43	10.17–10.69
Fibrous Sarcoma	6.63	6.42–6.84
Liposarcoma	6.27	6.07–6.48
Sarcoma, NOS	4.30	4.13–4.47
Kaposi Sarcoma	3.94	3.79–4.11
Chondrosarcoma	3.13	2.99–3.28
Osteosarcoma	2.97	2.83–3.11
Vascular Sarcoma	1.93	1.82–2.05
Ewing’s Sarcoma	1.63	1.54–1.74
Synovial Sarcoma	0.92	0.85–1.00
Notochordal Sarcoma	0.86	0.79–0.94
Unknown Sarcoma	0.80	0.73–0.87
Dermatological Sarcoma	0.47	0.41–0.52

* Muscle sarcoma includes rhabdomyosarcoma and leiomyosarcoma (For further information on each subtype, please refer to Supplementary Table S1).

**Table 2:** Average demographic characteristics of sarcoma patients in Canada between 1992 and 2010.

Sarcoma Subtype	Total No. ^a^	% of Total Sarcomas ^b^	% Male	% Female	Mean Age at Diagnosis ± SD, Years ^c^
Muscle Sarcoma	6165	24	41	59	53.9 ± 22.1
Fibrous Sarcoma	3910	15	59	41	62.8 ± 19.3
Liposarcoma	3705	14	58	42	58.3 ± 16.4
Sarcoma, NOS	2560	10	48	52	61.4 ± 19.3
Kaposi Sarcoma	2330	9	92	8	46.0 ± 15.6
Chondrosarcoma	1860	7	58	42	52.4 ± 18.4
Osteosarcoma	1435	6	53	47	37.2 ± 25.4
Vascular Sarcoma	1145	4	49	51	61.8 ± 18.4
Ewing’s Sarcoma	965	4	58	42	22.0 ± 14.8
Synovial Sarcoma	550	2	53	47	43.2 ± 20.5
Notochordal Sarcoma	520	2	57	43	55.6 ± 20.5
Unknown Sarcoma	480	2	49	51	38.1 ± 20.7
Dermatological Sarcoma	270	1	53	47	46.0 ± 22.6
	Total No. ^a^	<40 y ^a^	40–59 y ^a^	≥60 y ^a^	Mean age at diagnosis ± SD, years
Total Sarcomas	25,895	6480	7915	11,240	53.6 ± 21.7

^a^ Rounded to the nearest 5; ^b^ Rounded to the nearest percent; ^c^ Rounded to the nearest single decimal point.

**Table 3:** Analysis of the subtype of sarcoma most commonly seen in each age group.

Age Group (Years)	Total Sarcoma Cases ^a^	Most Common Type of Sarcoma
0–9	845	Muscle Sarcoma
10–19	1520	Osteosarcoma
20–29	1365	Ewing’s Sarcoma
30–39	2750	Kaposi Sarcoma
40–49	3840	Muscle Sarcoma
50–59	4075	Muscle Sarcoma
60–69	4170	Muscle Sarcoma
70–79	4290	Muscle Sarcoma
80–89	2450	Fibrous Sarcoma
90–99	330	Fibrous Sarcoma

^a^ Rounded to the nearest 5.

**Table 4:** Sarcoma mortality rates based on ICD-9 or ICD-10 codes. The ICD-9 or ICD-10 codes were used throughout this study to determine sarcoma mortality rates.

ICD Code	Study Code	Sarcoma Mortality
C40	C40 peripheral	Bone, joints, and articular cartilages of limbs located peripherally
C41	C41 axial	Bones, joints, and articular cartilages of other and unspecified sites located axially
C40–41	C40_41	Bones, joints, and articular cartilages of all locations combined
C49	C49 peripheral	Connective, subcutaneous, and other soft tissues located peripherally
C49	C49 axial	Connective, subcutaneous, and other soft tissues located axially
C49	C49	Connective, subcutaneous, and other soft tissues of all locations combined

**Table 5:** Mortality rates of axial and peripheral sarcomas in Canada between 1992 and 2010, presented as crude mortality rate per million individuals per year along with the upper and lower 95% CI.

Sarcoma	Mortality Rate
Axial Bone Sarcoma Peripheral Bone Sarcoma	1.35 (95% CI 1.25–1.44) 0.52 (95% CI 0.47–0.59)
Axial Soft Tissue Sarcoma Peripheral Soft Tissue Sarcoma	2.06 (95% CI 1.94–2.18) 1.21 (95% CI 1.12–1.30)

“CI” stands for “confidence interval”.

**Table 6:** Kaposi sarcoma incidence by socioeconomic status (SES).

	Quintile 1	Quintile 2	Quintile 3	Quintile 4	Quintile 5
Annual income	<CAD 20,000	CAD 20,000–25,000	CAD 25,000–30,000	CAD 30,000–35,000	>CAD 35,000
Cases ^a^	620	360	215	40	0
Incidence rate per million	3.40	1.67	1.64	0.89	0.00
Incidence rate ratio compared to Q1 (95% CI)	-	0.49 (0.43–0.56)	0.48 (0.41–0.56)	0.26 (0.19–0.36)	-

^a^ Cases are rounded to the nearest 5. Incidence rates are shown as cases per million individuals per year.

**Table 7:** Kaposi sarcoma incidence by African Canadian ethnicity.

	Quintile 1	Quintile 2	Quintile 3	Quintile 4	Quintile 5
Percentage of African Canadian individuals	<5.00%	5.00–9.99%	10.00–14.99%	15.00–19.99%	>20.00%
Cases ^a^	1045	95	35	30	20
Incidence rate per million	2.13	2.00	1.77	4.55	3.75
Incidence rate ratio compared to Q1 (95% CI)	-	0.94 (0.76–1.16)	0.83 (0.59–1.16)	2.14 (1.49–3.07)	1.76 (1.13–2.74)

^a^ Cases are rounded to the nearest 5. Incidence rates are shown as cases per million individuals per year.

**Table 8:** Kaposi sarcoma incidence by Hispanic ethnicity.

	Quintile 1	Quintile 2	Quintile 3	Quintile 4	Quintile 5
Percentage of Hispanic individuals	<1.00%	1.00–1.99%	2.00–2.99%	3.00–3.99%	>4.00%
Cases ^a^	220	480	310	155	60
Incidence rate per million	0.54	4.70	9.48	14.67	3.80
Incidence rate ratio compared to Q1 (95% CI)	-	8.74 (7.45–10.25)	17.61 (14.81–20.93)	27.25 (22.19–33.47)	7.06 (5.31–9.39)

^a^ Cases are rounded to the nearest 5. Incidence rates are shown as cases per million individuals per year.

## Data Availability

All available data are presented in the paper and are publicly available form the Canadian Cancer Registry and the Registre Québécois du Cancer (LRQC).

## Supplementary Materials

The following supporting information can be downloaded at https://www.mdpi.com/article/10.3390/curroncol30060424/s1. The ICD-O3 sarcoma subtypes included in this study are outlined in Supplementary Table S1.
