# Supplementary material for: Investigation of the Incidence and Geographic Distribution of Bone and Soft Tissue Sarcomas in Canada: A National Population-Based Study
Source: Curr Oncol. 2023 Jun 9;30(6):5631–51. doi: 10.3390/curroncol30060424 (PMC10297017; doi:10.3390/curroncol30060424)
Supplement: Supplementary file 1 [file curroncol-30-00424-s001.zip › curroncol-2329889-supplementary.pdf]

SUPPLEMENTARY TABLE S1. ICD-O-3 sarcoma subtypes in each group outlined in this study.

Group	ICD-O-3 and subtype
Sarcoma, not otherwise specified (NOS)	8800/3 Sarcoma, not otherwise specified (NOS)
Kaposi Sarcoma	9140/3 Kaposi sarcoma
Chondrosarcoma	9220/3 Chondrosarcoma, NOS<br>9221/3 Chondrosarcoma, Juxtacortical<br>9221/3 Chondrosarcoma, Periosteal<br>9243/3 Chondrosarcoma, Dedifferentiated<br>9240/3 Chondrosarcoma, Mesenchymal<br>9242/3 Chondrosarcoma, Clear cell<br>9220/3 Fibrochondrosarcoma<br>9231/3 Myxoid chondrosarcoma
Fibrous	8810/3 Fibrosarcoma, NOS<br>8814/3 Infantile fibrosarcoma<br>8830/3 Malignant fibrous histiocytoma<br>8806/3 Desmoplastic small round cell tumor
Synovial	9040/3 Synovial Sarcoma, NOS<br>9044/3 Clear Cell sarcoma, NOS<br>9044/3 Clear cell sarcoma, of tendons and aponeuroses<br>9044/3 Melanoma, malignant, of soft parts
Osteosarcoma	9180/3 Osteosarcoma, not otherwise specified (NOS)<br>9183/3 Osteosarcoma, Telangiectatic<br>9185/3 Osteosarcoma, Small cell<br>9187/3 Osteosarcoma, interosseous well differentiated<br>9192/3 Osteosarcoma, Parosteal<br>9193/3 Osteosarcoma, Periosteal<br>9194/3 Osteosarcoma, High grade surface
Notochord	9370/3 Chordoma, NOS<br>8990/3 Mesenchymoma, malignant
Unknown sarcoma	9364/3 Peripheral neuroectodermal tumor<br>9364/3 Neuroectodermal tumor, NOS<br>9364/3 Peripheral primitive neuroectodermal tumor, NOS
Ewing’s sarcoma	9260/3 Ewing’s sarcoma
Vascular	9120/3 Hemangiosarcoma<br>9133/3 Epithelioid haemangioendothelioma, malignant<br>9133/3 intravascular bronchial alveolar tumor
Liposarcoma	8851/3 Liposarcoma, well differentiated<br>8851/3 Liposarcoma, sclerosing<br>8851/3 Liposarcoma, inflammatory<br>8858/3 Liposarcoma dedifferentiated<br>8852/3 Liposarcoma, myxoid<br>8853/3 Liposarcoma, round cell<br>8854/3 Liposarcoma, pleomorphic<br>8855/3 Liposarcoma, mixed<br>8850/3 Liposarcoma, NOS
Muscle	8890/3 Leiomyosarcoma, NOS

	8910/3 Embryonal rhabdomyosarcoma, NOS<br>8910/3 Embryonal rhabdomyosarcoma, pleomorphic<br>8912/3 Spindle cell rhabdomyosarcoma<br>8910/3 sarcoma botryoides<br>8920/3 Alveolar rhabdomyosarcoma<br>8901/3 Pleomorphic rhabdomyosarcoma, adult type<br>8901/3 Pleomorphic rhabdomyosarcoma, NOS<br>9581/3 Alveolar soft part sarcoma<br>8963/3 Malignant rhabdoid tumor
Dermatological	8711/3 Glomus tumor, malignant<br>8804/3 Epithelioid sarcoma
